# The efficacy of a low-dose combination of febuxostat and benzbromarone versus each drug used alone for the treatment of gout

**DOI:** 10.3389/fmed.2025.1469879

**Published:** 2025-02-07

**Authors:** Caiyu Zheng, Qingwen Tong, Zhijun Zhang, Chunmei Lin, Zhiyi Wang, Dongshu Kang, Yanmei Lin, Jianqing Tian

**Affiliations:** ^1^Fujian Medical University Xiamen Humanity Hospital, Xiamen, China; ^2^School of Medicine, Xiamen University, Xiamen, China; ^3^The School of Clinical Medicine, Fujian Medical University, Fuzhou, China

**Keywords:** febuxostat, benzbromarone, gout, 24-h uric acid, uric acid

## Abstract

**Objective:**

The study focuses on comparing the efficacy of a low-dose combination of febuxostat and benzbromarone versus each drug used alone for the treatment of gout.

**Methods:**

A prospective, randomized, open-labeled trial of men with gout and renal uric acid underexcretion was conducted. We randomly assigned 100 patients to either low-dose febuxostat or low-dose benzbromarone for initial treatment. 43 patients with complete data were analyzed in this study. The analysis of medication Treatment effects, Period effects and Patient-Within-Sequence effects for different treatment options was performed by cross-test ANOVA.

**Result:**

The cross-trial analysis revealed no significant differences in the magnitude of uric acid decline among the three groups after treatment. Similarly, there were no statistically significant differences in blood lipid levels, CRP, CA724, eGFR, and ALT among the three groups post-treatment. The null model without participant-specific benefits was a linear mixed model for ALT, AST, eGFR, TC, TG, LDL, UA, CRP, CA724, and 24-h uric acid with age, BMI, treatment period and treatment as fixed factors, and the intercept as a random factor by participant. The results indicated no significant differences on relevant indicators among the three treatment regimens.

**Conclusion:**

Our study did not identify a difference in the efficacy of reducing uric acid excretion in gout patients when comparing conventional dose febuxostat and benzbromarone monotherapy versus low-dose combination therapy.

## Introduction

Over the last few decades, gout has become a substantial burden on human health and the global economy. Current evidence suggests it is closely linked to chronic kidney disease (CKD), stroke, and cardiovascular diseases (CVD) (1). Patients with uncontrolled gout experience higher indirect and total costs compared to those with controlled gout (2). The incidence of gout increases with elevated levels of hyperuricemia (3). An increasing body of evidence suggests that the pathophysiology of hyperuricemia is heterogeneous in gout patients (4–7). Renal uric acid underexcretion is the dominant cause of hyperuricemia (70–90% of gout patients) (5). Hence, criteria such as fractional excretion of uric acid (FEUA) less than 5.5% and UUE less than or equal to 600 mg/day/1.73 m2 are employed to define the subset of gout associated with renal uric acid underexcretion (7).

Medications designed for gout function as anti-inflammatory agents to address acute arthritis or as urate-lowering agents to manage hyperuricemia. Urate-lowering therapy (ULT) stands as the central approach for effectively controlling hyperuricemia and gout (8, 9). ULT operates through two mechanisms: promoting urate excretion and inhibiting urate production. Uricosurics contribute to the former, while xanthine oxidase (XO) inhibitors are involved in the latter (10). In China, the most frequently employed urate-lowering drugs include Allopurinol, Febuxostat, and benzbromarone.

Within the Asian study population, the prevalence of HLA-B*5801 is reportedly higher in individuals experiencing Allopurinol hypersensitivity, particularly among the Han and Korean populations (7.4%) (11–14). In Europe, benzbromarone is only recommended as a second-line ULT agent due to potential hepatotoxic effects (15).

It has been suggested that patients taking benzbromarone alone or combined allopurinol and benzbromarone therapy achieve a faster reduction in urate levels compared to those taking allopurinol alone. Some experts argue that the primary focus should be on comparing allopurinol and placebo. Additionally, they highlight allopurinol versus febuxostat and versus benzbromarone as the most clinically relevant active comparisons, and recommend restricting reporting to these comparisons (16).

This study aimed to refine the classification of hyperuricemia causes and select appropriate urate-lowering drugs based on the underlying pathogenesis. This approach seeks to improve patient compliance with uric acid management and gout treatment by utilizing smaller or conventional drug doses. While benzbromarone and Febuxostat have been employed in gout treatment, comparative studies assessing their urate-lowering effects have yielded conflicting results. Therefore, this study focuses on comparing the efficacy of a low-dose combination of febuxostat and benzbromarone versus each drug used alone for the treatment of gout.

## Materials and methods

A total of 100 gout patients exhibiting reduced uric acid excretion and receiving treatment at the Outpatient Department of Endocrinology, Fujian Medical University Xiamen Humanity Hospital, Xiamen, Fujian Province, from October 2019 to June 2023, were included in the present study. Our study included patients who were primarily middle-aged and young men with mild gout conditions, and there was no significant tophi formation or other severe gout manifestations in our subjects. These patients had mild gout attacks and had not been regularly treated to manage uric acid levels. Inclusion criteria comprised individuals aged 18 to 60, male gender, primary gout with serum uric acid levels ranging from 480 μmol/L to 600 μmol/L, uric acid excretion fraction <5.5%, 24-h uric acid ≤600 mg/d, and morning urine pH < 6.0. Additionally, participants should not have experienced an acute gouty arthritis attack in the 2 weeks preceding inclusion and during the 2-week washout period. Volunteers were required to sign an informed consent form. Exclusion criteria included severe diseases affecting blood, immune system, central nervous system, etc.; malignant tumors; acute gouty arthritis caused by chemoradiotherapy, drugs, or other factors; severe inflammatory reactions in other body parts; and the use of drugs affecting serum uric acid metabolism during treatment. The participants were classified using a random number method, and all participants provided written informed consent. The study was approved by Medical Ethics Committee of Xiamen Humanity Hospital (Approval NO. HAXM-MEC-20200608-006-01).

**Figure 1:**
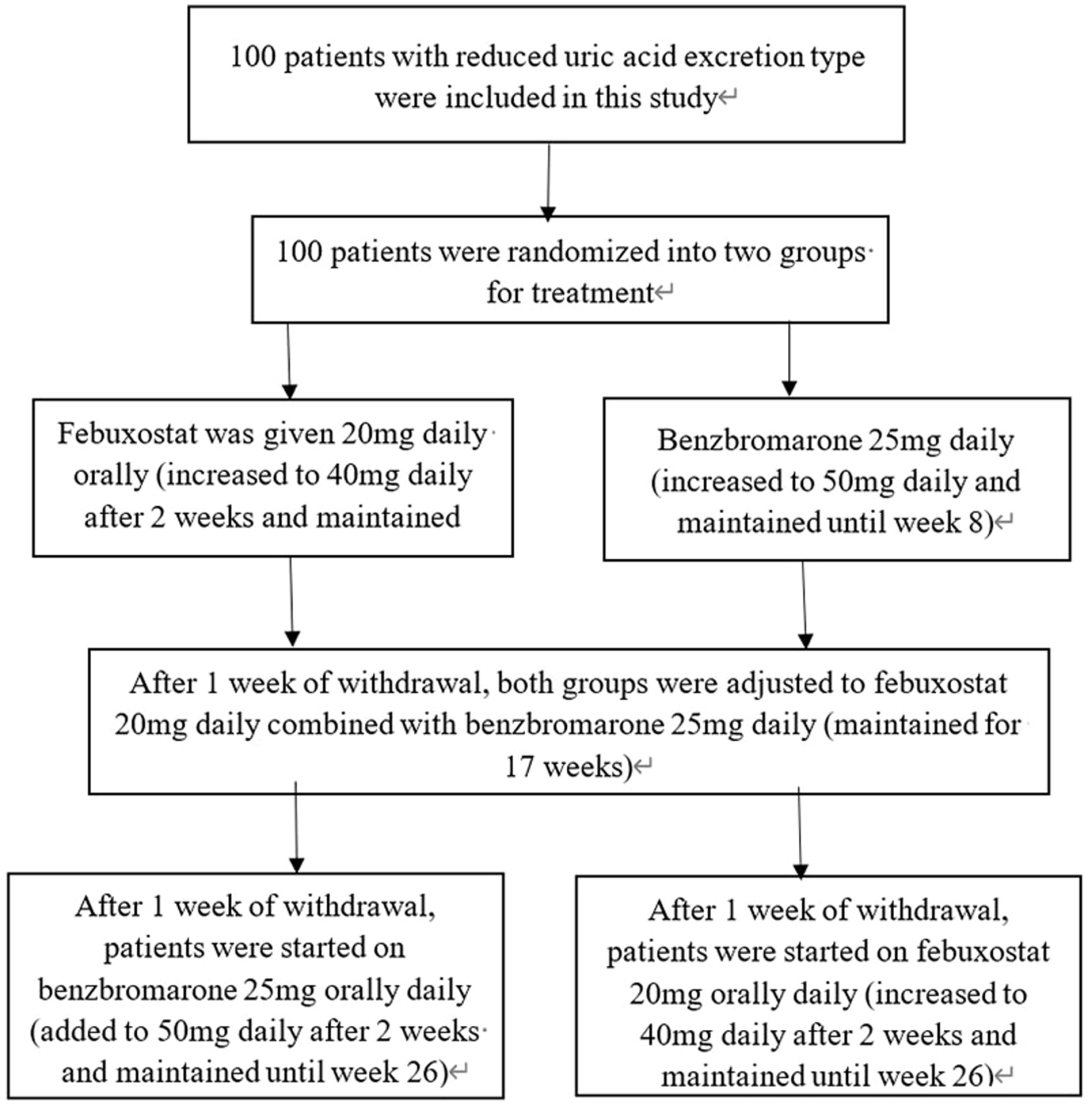
Flowchart.

In this study, the randomization principle was employed to assign patients meeting the enrollment criteria to either treatment A or treatment B. Treatment A involved a low-dose regimen of febuxostat (20 mg febuxostat once daily), while treatment B commenced with a low dose of benzbromarone, specifically 25 mg of benzbromarone once daily. Treatment A + B involved a combination of low-dose benzbromarone and low-dose febuxostat, i.e., 20 mg of febuxostat once daily combined with 25 mg of benzbromarone once daily to reduce uric acid. All participants underwent a 14-day washout period during which they refrained from taking uric acid-lowering medications, other medications, and adhered to a low-purine diet. Throughout the study, the use of other uric acid-lowering drugs or medications known to impact serum uric acid levels was prohibited. Participants were required to orally consume sodium bicarbonate daily to alkalize their urine. Exclusion criteria included patients who did not take medicine for three consecutive days or did not attend regular reviews. The study spanned 26 weeks, during which patients underwent eight examinations and follow-up visits at the end of the 2nd, 4th, 8th, 13th, 17th, 20th, 22nd, and 26th weeks after treatment at the gout specialized clinic. Anthropometric and biochemical measurements, including age, body weight, height, uric acid, alanine aminotransferase (ALT), aspartate aminotransferase (AST), triglyceride (TG), cholesterol (TC), CRP (C-reactive protein), and eGFR (Estimating the glomerular filtration rate), were collected at each follow-up (Figure 1). In the present study, no patient discontinued treatment due to drug intolerance or other adverse reactions. Finally, 43 patients completed the follow-up.

A modified cross-design method was utilized, and mixed-effect models were employed to account for confounding factors. IBM SPSS Statistics 21.0 was used for data analysis. Continuous variables were expressed as mean ± standard deviation. Drug main effects, phase effects, and sequential effects were analyzed using a multifactor mixed-effect model, with treatment, age, and BMI as fixed factors and the intercept as random factors for participants. Models were fitted using the maximum-likelihood method, and *p*-values were obtained through parameter bootstrap with 10,000 iterations.

## Result

In total, 43 patients were included in the current study, and Table 1 presents their baseline characteristics. The mean age in this small cohort was 33.33 ± 8.33 years. Additionally, the mean BMI, mean eGFR, and mean uric acid level for these 43 patients were 27.80 ± 3.26 kg/m^2^, 99.54 ± 12.34 mL/min/1.73m^2^, and 563.97 ± 92.40 μmol/L, respectively.

**Table 1:** Demographic and baseline clinical characteristics.

	Randomized participants (*N* = 43)
Age, mean (SD), years	33.33 (8.33)
BMI, mean (SD), kg/m^2^	27.80 (3.26)
ALT, mean (SD), U/L	46.87 (36.28)
AST, mean (SD), U/L	29.43 (16.80)
eGFR, mean (SD), ml/min/1.73m^2^	99.54 (12.35)
TC, mean (SD), mmol/L	5.35 (0.93)
TG, mean (SD), mmol/L	2.35 (1.34)
LDL, mean (SD), mmol/L	3.52 (0.72)
UA, mean (SD), μmol/L	563.97 (92.39)
CRP, mean (SD), mg/L	3.86 (5.11)
CA724, mean (SD), U/mL	4.30 (5.94)
24-h urine uric acid, mean (SD), μmol/day	1817.23 (859.05)

Data were presented as mean ± SD or median (interquartile ranges) for continuous variables, and numbers (proportions) for categorical variables. UA, uric acid; TG, triglycerides; TC, total cholesterol; HDL-c, high density lipoprotein cholesterol; LDL, low density lipoprotein cholesterol; eGFR, estimated glomerular filtration rate; CRP, C-reactive protein.

Table 2 displays the results of the cross-test analysis, with randomization used for grouping. Cross-trial ANOVA for different treatment options. Group A received doses of febuxostat, group A + B received a combination of low-dose febuxostat and low-dose benzbromarone, and group B underwent benzbromarone therapy. The cross-trial analysis revealed no significant differences in the magnitude of uric acid decline among the three groups after treatment. Similarly, there were no statistically significant differences in blood lipid levels, CRP, CA724, eGFR, and ALT among the three groups post-treatment.

**Table 2:** *p* Values of main effects and interaction effects between treatment and stage on outcome measures.

Outcome	Treatment	Mean (SD)	Treatment effects test		Period effects test		Sequence effects test	
			F	*P*	F	*P*	F	*P*
UA	A	398.42(112.93)	1.05	0.35	0.57	0.45	0.58	0.44
	A + B	368.34(117.28)						
	B	367.51(96.00)						
TG	A	2.57(1.79)	0.01	0.99	0.02	0.89	12.4	0.01
	A + B	2.52(2.26)						
	B	2.52(1.55)						
TC	A	5.20(0.79)	0.35	0.7	0.12	0.72	3.92	0.05
	A + B	5.13(1.04)						
	B	5.31(1.05)						
LDL	A	3.30(0.67)	0.34	0.71	0.17	0.67	0.09	0.76
	A + B	3.38(0.70)						
	B	3.43(0.76)						
eGFR	A	100.59(12.93)	0.28	0.76	0.06	0.82	1.69	0.19
	A + B	99.23(12.77)						
	B	101.36(12.80)						
CRP	A	2.75(3.28)	1.77	0.17	0.19	0.66	1.03	0.31
	A + B	4.73(7.10)						
	B	3.23(3.33)						
AST	A	36.45(36.98)	1.43	0.24	0.77	0.38	2.04	0.16
	A + B	31.63(15.97)						
	B	27.16(14.04)						
24-h urine UA	A	1852.11(1223.41)	0.81	0.45	2.63	0.11	0.08	0.77
	A + B	1775.92(1629.16)						
	B	2142.79(1212.65)						

The analysis of medication Treatment effects, Period effects and Patient-Within-Sequence effects for different treatment options was performed by cross-over ANOVA.

To further account for confounding factors, a mixed-effects model analysis was conducted. The effects of different treatment options on relevant indicators were analyzed using a multifactor mixed effect model. The null model without participant-specific benefits was a linear mixed model for ALT, AST, eGFR, TC, TG, LDL, UA, CRP, CA724, and 24-h uric acid with age, BMI, treatment period and treatment as fixed factors, and the intercept as a random factor by participant. Treatment, age, and BMI were considered fixed factors, while the intercept served as the random factor for participants. The results indicated no significant differences on relevant indicators among the three treatment regimens (Table 3).

**Table 3:** Results of the linear mixed-effect model analysis for assessing treatment on outcome variables.

Outcome variables	Estimate of treatment	95% Confidence interval		*P* value
		Lower bound	Upper bound	
ALT, mean (SD), U/L	−12.83	−167.33	141.67	0.87
AST, mean (SD), U/L	−4.84	−10.18	0.49	0.08
eGFR, mean (SD), ml/min/1.73m^2^	0.32	−2.31	2.95	0.81
TC, mean (SD), mmol/L	0.06	−0.15	0.26	0.60
TG, mean (SD), mmol/L	−0.01	−0.39	0.36	0.96
LDL, mean (SD), mmol/L	0.06	−0.09	0.21	0.41
UA, mean (SD), μmol/L	−16.12	−39.23	6.99	0.17
CRP, mean (SD), mg/L	0.25	−0.73	1.23	0.61
CA724, mean (SD), U/mL	−3.01	−59.09	53.07	0.92
24-h urine UA, mean (SD), μmol/day	119.07	−180.18	418.31	0.43

The null model without participant-specific benefits was a linear mixed model for ALT, AST, eGRR2, TC2, TG2, LDL2, UA, CRP, CA724, and 24-h uric acid with age, BMI, treatment period and treatment as fixed factors, and the intercept as a random factor by participant.

## Discussion

In our study, no significant difference in efficacy was observed among patients with gout and reduced uric acid excretion when comparing conventional-dose monotherapy with febuxostat and benzbromarone versus low-dose combination therapy.

During clinical practice, a significant portion of patients with hyperuricemia or gout, currently receiving urico-synthesis inhibitors, often do not undergo comprehensive testing, such as 24-h urinary uric acid excretion and uric acid excretion fraction. In both domestic and foreign studies on hyperuricemia or gout treatment, there has been a lack of classification based on the causes of hyperuricemia, leading to a limited understanding of the condition. Existing research suggests that although approximately 70% of gout patients in China have received treatment, the compliance rate for uric acid management is only around 10%. Moreover, only 20% of individuals treated for more than 6 months exhibit regular adherence, indicating a significant challenge in maintaining a satisfactory compliance rate (17).

Earlier studies, including the work of scholars like Perez-Ruiz et al. (18), have suggested that in gout patients with low uric acid excretion, the uric acid excretion drug benzbromarone can achieve lower uric acid levels than allopurinol, a uric acid synthesis inhibitor. Notably, the efficacy of febuxostat 40 mg is comparable to that of allopurinol (11). Research by Ohta Yuko et al. (19) and Kojima et al. (20) suggested that combining febuxostat, a low-dose uric acid synthesis inhibitor, with benzbromarone, a uricoproexcretion drug, yields a significantly greater effect on lowering blood uric acid than conventional doses of febuxostat or benzbromarone alone. Other investigations have indicated that patients using febuxostat exhibit higher estimated glomerular filtration rates, a reduced risk of kidney disease progression, and lower serum uric acid levels compared to those using allopurinol, suggesting potential renal protective benefits of febuxostat. In China, studies have shown that febuxostat is more effective in treating hyperuricemia than benzbromarone, effectively reducing uric acid levels and demonstrating a higher safety profile (21, 22). Additionally, previous findings suggest that lower doses of benzbromarone have a greater impact on lowering serum uric acid compared to lower doses of febuxostat (23).

However, the present study did not identify a difference in uric acid reduction between febuxostat and benzbromarone, whether used as conventional dose monotherapy or in low-dose combination therapy. Notably, we found that both monotherapy and low-dose treatments exhibited equal effectiveness. These findings align with a recent study that demonstrated comparable efficacy between benzbromarone (25 mg/d) and febuxostat (20 mg/d) in reducing serum urate levels (24). In addition, Nan et al. emphasized the significance of considering uric acid levels and renal function when recommending low doses of febuxostat and benzbromarone to patients with gout. Their research indicates that the efficacy of both medications is influenced by renal function.

Other factors that may influence the results include: First, we provided simple lifestyle intervention guidance to each study subject, but the degree of adherence to lifestyle management may vary among individuals, which could affect the measurement of uric acid levels. Second, the follow-up period for this study was relatively short, and the dosages were not further increased to full dose across groups. Further exploration of the results after increasing the dosage is warranted. Our study findings align with existing guidelines. Despite the various types of hyperuricemia, both domestic and international guidelines currently do not recommend tailoring uric acid-lowering therapy based on specific types. Due to reports of hepatocellular necrosis associated with benzbromarone in Caucasians, it is often recommended as a second-line drug in European guidelines. The American College of Rheumatology (ACR) and the European League Against Rheumatism (EULAR) recommend benzbromarone at 50 mg once daily, gradually increasing to 100 mg daily and the 2012 ACR recommends that clinicians consider the cause of hyperuricemia for gout patients (evidence level C) (25). However, the ACR’s latest updated gout management guidelines conditionally advise against checking urine for drug selection accuracy and uric acid adjuvant therapy strategy (26). The 2016 European Congress of Rheumatology (EULAR) Gout Management Guidelines and the 2020 ACR guidelines recommend allopurinol as a first-line treatment. While the 2016 EULAR guidelines support uric acid excretion as a second-line treatment for gout, the 2020 ACR guidelines conditionally recommend probenecid as a second-line agent only after allopurinol has failed, and benzbromarone is not part of this clinical guideline due to its unavailability in the United States (27, 28).

Our research distinguishes itself by focusing on a specific advantage: while numerous clinical trials and studies globally intervene directly or analyze the causes of hyperuricemia without categorizing patients into types, our study uniquely targets gout patients with low uric acid excretion. Through distinct group trials, we administered two conventional doses of uric acid-lowering drugs with varying mechanisms of action as standalone treatments for individuals with low uric acid excretion. Additionally, the treatment group explored the effectiveness of different treatment regimens by incorporating a combination of low doses of two uric acid-lowering drugs with distinct mechanisms of action. It is essential to acknowledge some limitations, such as the study being exclusively conducted in males and our study primarily focused on comparing the efficacy of drugs between different groups and did not include analyses of the differences in the timing and duration of gout attacks between groups. We did not further increase the drug dosage in each group to explore the differences in efficacy following dose escalation. Future research with a larger sample size is needed to validate and further confirm our findings.

In conclusion, our study did not identify a difference in the efficacy of reducing uric acid excretion in gout patients when comparing conventional dose febuxostat and benzbromarone monotherapy versus low-dose combination therapy.

## Data Availability

The original contributions presented in the study are included in the article/supplementary material, further inquiries can be directed to the corresponding author.
